# Response to Fergusson & Boden (2015): The importance of considering the impacts of survey non‐participation

**DOI:** 10.1111/add.13016

**Published:** 2015-07-29

**Authors:** Anne Illemann Christensen, Ola Ekholm, Linsay Gray, Charlotte Glümer, Knud Juel

**Affiliations:** ^1^ National Institute of Public HealthUniversity of Southern DenmarkCopenhagenDenmark; ^2^ MRC/CSO Social and Public Health Sciences UnitUniversity of Glasgow, Glasgow, UK and Department of Epidemiology, Columbia UniversityNew YorkNYUSA; ^3^ Research Centre for Prevention and HealthThe Capital Region of DenmarkGlostrupDenmark

**Keywords:** Bias, health behaviour, morbidity, mortality, non‐response

We thank Fergusson & Boden for their interest in our paper, as detailed in their commentary 1. They point to some interesting issues which, for the main part, have also been debated during the preparation of the paper.

The first issue raised is that with the large sample size, the likelihood of finding significant group differences is high. We acknowledge that this should be considered, but as the hazard ratios in the present study are relatively large and confidence limits in most cases relatively narrow, we consider the large sample size to be more a strength than a drawback.

Ferguson & Boden also point to the fact that the low baseline rates of morbidity and mortality will generate relatively large hazard ratios, even with relatively small differences in the absolute number of events between respondents and non‐respondents. We acknowledge that looking only at relative differences can be somewhat misleading, and we have therefore also provided the absolute number of events and the rates for each group in our paper 2. The most careful way of interpreting results is often to look at both relative and absolute differences. However, as pointed out by Ferguson & Boden, the use of respondent‐only data can cause biased estimates even when the absolute difference in the number of events among respondents and non‐respondents is small.

It is suggested that the future sample sizes could be reduced and the saved cost should be used on contacting the non‐contacts. This is an interesting reflection. Consideration of strategies to raise the response rate among specific groups of non‐respondents is indeed warranted, and different strategies to improve response rate among different types on non‐response groups are presented in our paper 2. However, the present study used pooled data from two health surveys. Hence, the sample size in each of the surveys is not as large as it might appear in the commentary by Fergusson & Boden. Furthermore, both surveys are designed to provide county and regional representative data, respectively, and hence a minimum sample size is required in each county/region. The number of non‐contacts is very small in the present study, thanks to a notable effort to establish contact with all invited individuals, and we think that it would be very difficult to establish contact with all invited individuals even if the resources are used differently. However, their suggestion will be considered when planning future surveys.

Lastly, Ferguson & Boden argue that non‐response bias may have less of an impact when examining exposure–outcome associations compared to studies of prevalence estimates. This is often true 3, 4, 5. There are, however, exceptions, and thus the impact of non‐response bias in studies of associations cannot be assumed to be negligible 6.

In summary, Ferguson & Boden raise some interesting issues, which we agree need to be taken into account when both analysing and interpreting results on non‐response in surveys, while the impact of survey non‐participation should not be overlooked.

## Declaration of interest

None.
